# A Comparison of Frailty Indices in Predicting Postoperative Outcomes After Endoscopic Sinus Surgery

**DOI:** 10.1002/alr.70212

**Published:** 2026-07-02

**Authors:** Akshay Warrier, David Remyes, Tenesha Boyd, Ailish Nagpal, Aman M Patel, David W. Wassef, Jean Anderson Eloy

**Affiliations:** ^1^ Department of Otolaryngology‐Head and Neck Surgery Rutgers New Jersey Medical School Newark New Jersey USA; ^2^ Department of Osteopathic Manipulative Medicine College of Osteopathic Medicine New York Institute of Technology Old Westbury New York USA; ^3^ Department of Otolaryngology Meharry Medical College Nashville Tennessee USA; ^4^ Department of Otolaryngology Aram V. Chobanian & Edward Avedisian School of Medicine Boston University Boston Massachusetts USA; ^5^ Center for Skull Base and Pituitary Surgery Neurological Institute of New Jersey Rutgers New Jersey Medical School Newark New Jersey USA

## Abstract

**Objective:**

To compare the discriminatory performance of the Risk Analysis Index (RAI) and modified Frailty Index‐5 (mFI‐5) in predicting postoperative outcomes following endoscopic sinus surgery (ESS).

**Study Design:**

Retrospective cohort study.

**Methods:**

Adult ESS patients were identified from the ACS‐NSQIP database (2005–2020). Frailty was quantified using the RAI and mFI‐5. Multivariable logistic regression evaluated associations between frailty strata and postoperative outcomes, including Clavien–Dindo (CD) complications, extended length of stay (eLOS), non‐home discharge (NHD), wound complications, and mortality. Model discrimination was assessed using receiver operating characteristic analysis with area under the curve (AUC) as the primary comparative metric.

**Results:**

Among 1871 patients, increasing frailty was independently associated with adverse postoperative outcomes across both indices. However, discriminatory performance differed meaningfully. The RAI demonstrated superior discrimination for NHD (AUC 0.796 vs. 0.721), CD II complications (0.722 vs. 0.547), and mortality (0.742 vs. 0.684), and CD IV (0.737 vs. 0.656), with comparable performance for eLOS (0.647 vs. 0.636). Overall, the RAI more consistently separated patients at risk for recovery‐ and disposition‐related endpoints.

**Conclusion:**

Frailty is a significant predictor of postoperative outcomes after ESS. While both indices identified elevated risk, the RAI demonstrated stronger and more consistent discrimination across clinically meaningful endpoints, particularly those reflecting postoperative recovery and resource utilization. Multidomain frailty assessment may therefore provide more informative risk stratification than comorbidity‐based tools in elective rhinologic surgery.

## 1 Introduction

Frailty, broadly defined as diminished physiologic reserve and reduced resistance to stressors, has been identified as a major determinant of postoperative outcomes across surgical specialties [1, 2, 3, 4, 5]. In contrast to chronological age alone, frailty reflects the cumulative effects of comorbidity burden, functional dependence, and cognitive impairment, providing a more patient‐centered estimate of surgical vulnerability [6, 7, 8]. As surgical populations age and value‐based care increasingly prioritizes accurate risk prediction and complication mitigation, preoperative frailty assessment has become central to operative decision‐making, counseling, and perioperative planning [9, 10].

Within otolaryngology, endoscopic sinus surgery (ESS) is routinely performed for chronic rhinosinusitis and related sinonasal conditions [11, 12]. Although ESS is generally considered a low‐to‐moderate risk operation, postoperative morbidity, prolonged recovery, and non‐home discharge (NHD) remain clinically meaningful outcomes. This is particularly true among medically complex patients [13, 14]. Importantly, even modest perioperative stressors may precipitate adverse outcomes in individuals with limited physiologic reserve, underscoring the importance of identifying frailty beyond traditional measures such as age or isolated comorbidities [7, 15, 16]. Despite this need, the optimal frailty tool for risk stratification in ESS remains incompletely defined [17].

Multiple frailty indices have been proposed for surgical risk prediction, the foremost among which are the modified Frailty Index‐5 (mFI‐5) and the Risk Analysis Index (RAI) [18, 19, 20]. The mFI‐5 offers simplicity and feasibility through a linear, five‐comorbidity‐based score, making it attractive for broad implementation [21]. However, its emphasis on comorbid conditions may underrepresent key frailty domains such as functional dependence and cognitive decline. In contrast, the RAI incorporates functional and cognitive measures alongside comorbidity burden, providing a more comprehensive proxy of physiologic reserve [22]. Across several surgical disciplines, the RAI has demonstrated stronger discrimination for postoperative morbidity, mortality, and discharge outcomes. However, evidence specific to otolaryngology, and ESS in particular, remains limited [22, 23, 24, 25, 26, 27, 28, 29].

Frailty has traditionally been emphasized in higher‐risk operations, leaving its implications for lower‐risk elective procedures comparatively undercharacterized. Accordingly, this study evaluates and compares the predictive performance of the RAI and mFI‐5 for postoperative outcomes following ESS using ACS‐NSQIP data. Clarifying the relative strengths of these indices in ESS may improve preoperative counseling, support individualized shared decision‐making, and inform targeted perioperative strategies for patients at greatest physiologic risk.

## 2 Methods

### 2.1 Study Design and Data Source

This was a retrospective cohort study using data from the American College of Surgeons National Surgical Quality Improvement Program (ACS‐NSQIP) database from 2005 to 2020. ACS‐NSQIP is a prospectively maintained, multi‐institutional registry capturing preoperative risk factors, intraoperative variables, and 30‐day postoperative outcomes across more than 700 participating hospitals. Because the database contains de‐identified patient information, this study was considered exempt from institutional review board approval.

### 2.2 Patient Selection

Adult patients (≥ 18 years) who underwent ESS were identified using relevant Current Procedural Terminology (CPT) codes (Table 1). Patients were included if complete data were available to calculate both the RAI and the mFI‐5. Those with missing frailty variables or incomplete outcome data were excluded from the analysis (Figure S1).

**TABLE 1:** Procedural CPT Codes for ESS‐Related Procedures.

CPT code	Procedure category
31237	Endoscopic nasal/sinus surgery with biopsy, polyp removal, or debridement
31239	Endoscopic nasal/sinus surgery with dacryocystorhinostomy (DCR)
31240	Endoscopic nasal/sinus surgery with concha bullosa resection
31254	Endoscopic ethmoidectomy, partial (anterior)
31255	Endoscopic ethmoidectomy, complete (anterior + posterior)
31256	Endoscopic maxillary antrostomy
31267	Endoscopic maxillary antrostomy with tissue removal from the maxillary sinus
31276	Endoscopic frontal sinus exploration, with or without tissue removal
31287	Endoscopic sphenoidotomy
31288	Endoscopic sphenoidotomy with tissue removal from the sphenoid sinus

### 2.3 Risk Analysis Index

The RAI is a validated frailty instrument incorporating demographic variables (age, sex), medical comorbidities (heart failure, renal impairment, dyspnea, cancer), nutritional status (weight loss), and functional performance, including activities of daily living. For this analysis, the retrospective administrative RAI (RAI‐A) was applied using ACS‐NSQIP variable definitions. Patients were stratified into four categories based on established thresholds: nonfrail (RAI 0–20), prefrail (RAI 21–30), frail (RAI 31–40), and severely frail (RAI ≥ 41).

### 2.4 Modified Frailty Index‐5

The mFI‐5 is derived from five ACS‐NSQIP variables: functional dependence, diabetes mellitus, chronic obstructive pulmonary disease, congestive heart failure, and hypertension requiring medication. Scores range from 0 to 5, with patients categorized as robust (0), prefrail (1), frail (2), or severely frail (≥ 3).

### 2.5 Outcomes

Postoperative outcomes assessed within 30 days included Clavien–Dindo (CD) Grade II, IIIb, and Grade IV complications, surgical site infection, wound dehiscence, extended length of stay (eLOS), NHD, and mortality. Exploratory analyses were performed for available NSQIP resource‐utilization outcomes, including 30‐day return to the operating room/reoperation and 30‐day readmission; however, postoperative ICU stay, and emergency department visits were not consistently available across the study period. CD Grade II complications were defined as complications requiring pharmacologic treatment, including blood transfusion or total parenteral nutrition. Grade IIIb complications were defined as interventions requiring general anesthesia, and Grade IV complications were defined as life‐threatening complications requiring ICU management of organ dysfunction. CD IIIa complications were not separately analyzed because non‐general‐anesthesia interventions are not consistently captured in NSQIP. eLOS was defined as hospitalization exceeding the 75th percentile for the cohort.

### 2.6 Statistical Analysis

Baseline characteristics were summarized using descriptive statistics. Univariate analyses assessed the association between frailty indices and postoperative outcomes. Multivariable logistic regression models were constructed to evaluate associations between frailty measures and outcomes, adjusting for relevant demographic and clinical covariates, including age, sex, race/ethnicity, BMI, transfer status, nonelective surgery status, and operative time. Model discrimination was assessed using receiver operating characteristic (ROC) curve analysis, with area under the curve (AUC) calculated for each frailty index across outcomes. Statistical significance was defined as *p* < 0.05. All analyses were performed using SPSS version 28.

## 3 Results

### 3.1 Study Population

A total of 1954 ESS cases were identified in the ACS‐NSQIP database from 2005 to 2020. Patients were excluded for missing variables, missing postoperative outcome data, or missing covariates. A total of 1871 patients remained in the final analytic cohort. The cohort was 52.4% male (*n* = 981) and 47.5% female (*n* = 889). Baseline demographic and clinical characteristics of the cohort are summarized in Table 2. The distribution of frailty categories differed between the RAI and mFI‐5, with 78.9% (*n* = 1476) of patients classified as nonfrail by RAI compared to 61.6% (*n* = 1153) classified as robust by mFI‐5.

**TABLE 2:** Baseline Characteristics of patients undergoing endoscopic sinus surgery, *n* = 1871.

			RAI categories				mFI‐5 categories		
Category	Totals *n* = 1871	Robust *n* = 1476	Normal *n* = 326	Frail *n* = 62	Severely frail *n* = 7	Robust *n* = 1153	Pre‐frail *n* = 490	Frail *n* = 208	Severely frail *n* = 20
**Demographics**									
Age, median (IQR), years	56 (22)	50 (19)	69 (12)	74 (14)	75 (16)	49 (22)	59 (19)	63 (16)	64 (16)
Male, *n* (%)	981	684 (46.34%)	247 (75.77%)	45 (72.58%)	5 (71.43%)	581 (50.39%)	279 (56.94%)	112 (53.85%)	9 (45.00%)
Race, *n* (%)									
White, *n* (%)	1186	906 (61.38%)	228 (69.94%)	46 (74.19%)	6 (85.71%)	734 (63.66%)	311 (63.47%)	130 (62.50%)	11 (55.00%)
Black, *n* (%)	212	183 (12.40%)	25 (7.67%)	3 (4.84%)	1 (14.29%)	88 (7.63%)	83 (16.94%)	36 (17.31%)	5 (25.00%)
Asian, *n* (%)	57	45 (3.05%)	12 (3.68%)	0 (0.00%)	0 (0.00%)	39 (3.38%)	14 (2.86%)	3 (1.44%)	1 (5.00%)
Hispanic, *n* (%)	245	215 (14.57%)	27 (8.28%)	3 (4.84%)	0 (0.00%)	170 (14.74%)	50 (10.20%)	23 (11.06%)	2 (10.00%)
Other^a^, *n* (%)	171	127 (8.60%)	34 (10.43%)	10 (16.13%)	0 (0.00%)	122 (10.58%)	32 (6.53%)	16 (7.69%)	1 (5.00%)
**Preoperative clinical status**									
Hypertension, *n* (%)	628	390 (26.42%)	205 (62.88%)	29 (46.77%)	4 (57.14%)	0 (0.00%)	408 (83.27%)	202 (97.12%)	18 (90.00%)
CHF, *n* (%)	13	2 (0.14%)	4 (1.23%)	6 (9.68%)	1 (14.29%)	0 (0.00%)	0 (0.00%)	6 (2.88%)	7 (35.00%)
COPD, *n* (%)	61	27 (1.83%)	22 (6.75%)	10 (16.13%)	2 (28.57%)	0 (0.00%)	19 (3.88%)	29 (13.94%)	13 (65.00%)
Dyspnea, *n* (%)	85	44 (2.98%)	29 (8.90%)	9 (14.52%)	3 (42.86%)	25 (2.17%)	23 (4.69%)	29 (13.94%)	8 (40.00%)
Renal failure, *n* (%)	1	0 (0.00%)	0 (0.00%)	0 (0.00%)	1 (14.29%)	0 (0.00%)	0 (0.00%)	1 (0.48%)	0 (0.00%)
Dialysis, *n* (%)	5	2 (0.14%)	2 (0.61%)	0 (0.00%)	1 (14.29%)	0 (0.00%)	2 (0.41%)	3 (1.44%)	0 (0.00%)
Disseminated cancer, *n* (%)	42	0 (0.00%)	0 (0.00%)	35 (56.45%)	7 (100.00%)	28 (2.43%)	7 (1.43%)	6 (2.88%)	1 (5.00%)
Weight loss, *n* (%)	28	5 (0.34%)	10 (3.07%)	10 (16.13%)	3 (42.86%)	14 (1.21%)	10 (2.04%)	3 (1.44%)	1 (5.00%)
Non‐home origin, *n* (%)	45	31 (2.10%)	7 (2.15%)	7 (11.29%)	0 (0.00%)	24 (2.08%)	7 (1.43%)	11 (5.29%)	3 (15.00%)
Functional status, *n* (%)									
Independent, *n* (%)	1826	1457 (98.71%)	310 (95.09%)	54 (87.10%)	5 (71.43%)	1143 (99.13%)	477 (97.35%)	193 (92.79%)	13 (65.00%)
Partial dependence, *n* (%)	27	5 (0.34%)	13 (3.99%)	7 (11.29%)	2 (28.57%)	0 (0.00%)	8 (1.63%)	13 (6.25%)	6 (30.00%)
Total dependence, *n* (%)	3	1 (0.07%)	1 (0.31%)	1 (1.61%)	0 (0.00%)	0 (0.00%)	2 (0.41%)	0 (0.00%)	1 (5.00%)
Elective surgery status, *n* (%)	1643	1298 (87.94%)	290 (88.96%)	50 (80.65%)	5 (71.43%)	1024 (88.81%)	436 (88.98%)	168 (80.77%)	15 (75.00%)
**Postoperative outcomes**									
Any wound complication, *n* (%)	24	18 (1.22%)	5 (1.53%)	1 (1.61%)	0 (0.00%)	18 (1.56%)	4 (0.82%)	2 (0.96%)	0 (0.00%)
SSI, *n* (%)	10	7 (0.47%)	3 (0.92%)	0 (0.00%)	0 (0.00%)	7 (0.61%)	1 (0.20%)	2 (0.96%)	0 (0.00%)
DSSI, *n* (%)	4	3 (0.20%)	0 (0.00%)	1 (1.61%)	0 (0.00%)	3 (0.26%)	1 (0.20%)	0 (0.00%)	0 (0.00%)
OSSI, *n* (%)	7	7 (0.47%)	0 (0.00%)	0 (0.00%)	0 (0.00%)	5 (0.43%)	2 (0.41%)	0 (0.00%)	0 (0.00%)
Dehisc., *n* (%)	4	1 (0.07%)	2 (0.61%)	1 (1.61%)	0 (0.00%)	4 (0.35%)	0 (0.00%)	0 (0.00%)	0 (0.00%)
Clavien–Dindo II, *n* (%)	56	32 (2.17%)	16 (4.91%)	8 (12.90%)	0 (0.00%)	30 (2.60%)	16 (3.27%)	10 (4.81%)	0 (0.00%)
Clavien–Dindo IIIb, *n* (%)	47	31 (2.10%)	14 (4.29%)	2 (3.23%)	0 (0.00%)	30 (2.60%)	13 (2.65%)	4 (1.92%)	0 (0.00%)
Clavien–Dindo IV, *n* (%)	26	15 (1.02%)	8 (2.45%)	3 (4.84%)	0 (0.00%)	9 (0.78%)	8 (1.63%)	7 (3.37%)	2 (10.00%)
Mortality, *n* (%)	5	3 (0.20%)	2 (0.61%)	0 (0.00%)	0 (0.00%)	2 (0.17%)	0 (0.00%)	3 (1.44%)	0 (0.00%)
eLOS, *n* (%)	43	26 (1.76%)	13 (3.99%)	3 (4.84%)	1 (14.29%)	18 (1.56%)	10 (2.04%)	12 (5.77%)	3 (15.00%)
Non‐home discharge, *n* (%)	32	11 (0.75%)	16 (4.91%)	5 (8.06%)	0 (0.00%)	10 (0.87%)	5 (1.02%)	14 (6.73%)	3 (15.00%)

Abbreviations: CHF, congestive heart failure; Dehisc, wound dehiscence; DSSI, deep incisional site infection; eLOS, extended length of stay; OSSI, organ/space surgical site infection; SSI, superficial incisional site infection.

^a^ Unknown/Native Hawaiian or Other Pacific Islander/American Indian or Alaska Native.

### 3.2 Risk Analysis Index

Higher RAI categories were independently associated with increased odds of postoperative morbidity. On multivariable analysis, patients classified as frail by RAI (31–40) demonstrated significantly increased odds of CD Grade II complications (OR 5.78, 95% CI 2.23–14.96, *p* < 0.001) and CD Grade IV complications (OR 4.34, 95% CI 1.11–16.97, *p* = 0.035). Severely frail patients (RAI ≥ 41) had markedly elevated odds of eLOS (OR 11.71, 95% CI 1.30–105.89, *p* = 0.029). Among patients categorized as prefrail or frail by RAI, the odds of NHD were further amplified (prefrail OR 5.99, 95% CI 2.56–14.04, *p* < 0.001; frail OR 10.20, 95% CI 3.20–32.51, *p* < 0.001) (Table 3).

**TABLE 3:** Multivariable associations between frailty indices and postoperative outcomes.

Outcome	RAI (0‐20) nonfrail	RAI (21–30) Prefrail	RAI (31–40) Frail	RAI (40+) Severely frail	mFI‐5 (1) Nonfrail	mFI‐5 (2) Prefrail	mFI‐5 (3) Frail	mFI‐5 (4) Severely frail
**Mortality**	*Ref*	2.79 (0.40–19.35), *p* = 0.299	—	—	*Ref*	—	3.38 (0.39–29.24), *p* = 0.269	—
**CD I**	*Ref*	2.34 (0.55–9.99), *p* = 0.252	—	—	*Ref*	0.23 (0.03–2.04), *p* = 0.186	0.88 (0.15–5.37), *p* = 0.893	—
**CD II**	*Ref*	1.84 (0.90–3.76), *p* = 0.097	**5.78 (2.23–14.96), *p* < 0.001**	—	*Ref*	0.95 (0.44–2.03), *p* = 0.892	1.47 (0.59–3.62), *p* = 0.409	—
**CD IIIb**	*Ref*	1.44 (0.72–2.88), *p* = 0.298	0.98 (0.22–4.36), *p* = 0.980	—	*Ref*	0.88 (0.42–1.83), *p* = 0.731	0.58 (0.18–1.82), *p* = 0.348	—
**CD IV**	*Ref*	1.65 (0.61–4.48), *p* = 0.324	**4.34 (1.11–16.97), *p* = 0.035**	—	*Ref*	1.70 (0.57–5.03), *p* = 0.340	2.04 (0.56–7.45), *p* = 0.283	12.30 (1.95–77.69), *p* = 0.008
**eLOS**	*Ref*	1.64 (0.76–3.56), *p* = 0.210	2.73 (0.76–9.90), *p* = 0.126	**11.71 (1.30–105.89), *p* = 0.029**	*Ref*	1.17 (0.49–2.82), *p* = 0.728	**3.00 (1.18–7.60), *p* = 0.021**	**10.81 (2.36–49.51), *p* = 0.002**
**NHD**	*Ref*	**5.99 (2.56–14.04), *p* < 0.001**	**10.20 (3.20–32.51), *p* < 0.001**	—	*Ref*	0.88 (0.28–2.94), *p* = 0.875	**4.85 (1.74–13.54), *p* = 0.003**	**20.03 (3.82–104.92), *p* < 0.001**
**SSI**	*Ref*	2.34 (0.55–9.99), *p* = 0.252	—	—	*Ref*	0.23 (0.03–2.04), *p* = 0.186	0.88 (0.15–5.37), *p* = 0.893	—
**DSSI**	*Ref*	—	5.57 (0.49–63.81), *p* = 0.167	—	*Ref*	—	—	—
**OSSI**	*Ref*	—	—	—	*Ref*	1.25 (0.19–8.12), *p* = 0.814	—	—
**Dehiscence**	*Ref*	4.25 (0.37–49.10), *p* = 0.247	10.79 (0.62–189.38), *p* = 0.104	—	*Ref*	0.66 (0.06–7.38), *p* = 0.732	—	—

### 3.3 Modified Frailty Index‐5

Frailty as measured by mFI‐5 was associated with select postoperative outcomes. Higher mFI‐5 scores were independently associated with eLOS (frail OR 3.00, 95% CI 1.18–7.60, *p* = 0.021; severely frail OR 10.81, 95% CI 2.36–49.51, *p* = 0.002) and NHD (frail OR 4.85, 95% CI 1.74–13.54, *p* = 0.003; severely frail OR 20.03, 95% CI 3.82–104.92, *p* < 0.001). The mFI‐5 severely frail category was also associated with CD Grade IV complications (OR 12.30, 95% CI 1.95–77.69, *p* = 0.008) (Table 3).

### 3.4 Discriminatory Performance of Frailty Indices

ROC curve analysis demonstrated differential discriminatory performance between frailty indices across postoperative outcomes (Figure 1; Table 4). The RAI demonstrated higher discrimination for NHD (AUC 0.796 vs. 0.721), CD Grade II complications (0.722 vs. 0.547), and mortality (0.742 vs. 0.684). Discrimination for eLOS was comparable between indices (RAI 0.647 vs. mFI‐5 0.636).

**FIGURE 1:**
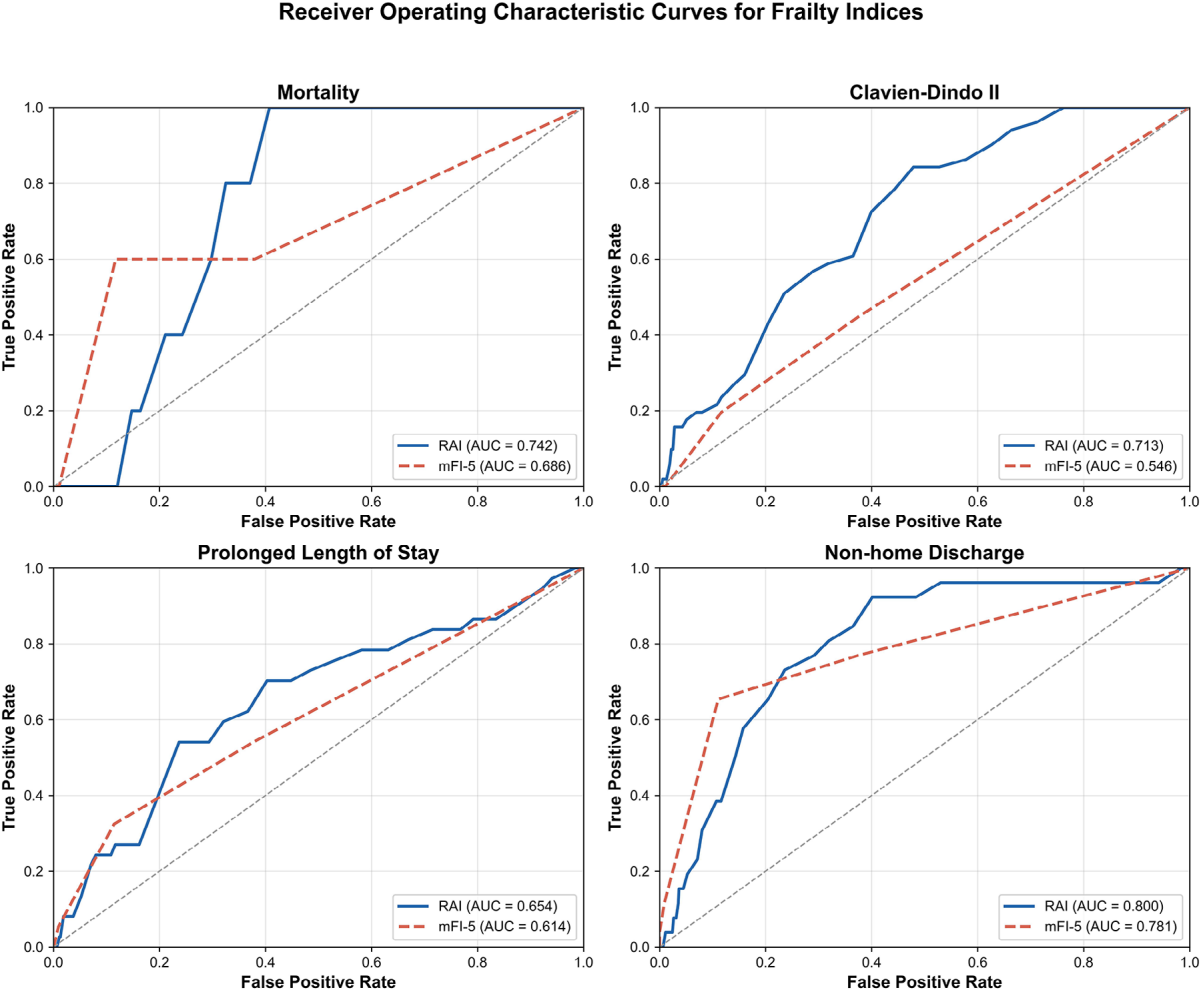
Receiver operating characteristic curves comparing RAI and mFI‐5 for postoperative outcomes after ESS legend: Receiver operating characteristic curves comparing the discriminatory performance of the Risk Analysis Index (RAI) and modified Frailty Index‐5 (mFI‐5) for postoperative outcomes after endoscopic sinus surgery. Outcomes shown include 30‐day mortality, Clavien–Dindo Grade II complications, prolonged length of stay, and non‐home discharge. Area under the curve values are displayed within each panel.

**TABLE 4:** Discriminatory performance (AUC) of frailty indices.

Outcome	RAI AUC (95% CI)	mFI‐5 AUC (95% CI)
Mortality	0.742 (0.661–0.823)	0.684 (0.397–0.972)
CD II	0.722 (0.667–0.776)	0.547 (0.469–0.626)
CD IV	0.737 (0.652–0.822)	0.656 (0.540–0.773)
eLOS	0.647 (0.560–0.734)	0.636 (0.542–0.730)
NHD	0.796 (0.724–0.869)	0.721 (0.616–0.827)

In contrast, the mFI‐5 demonstrated modestly higher discrimination for CD Grade IV complications (0.656 vs. 0.737). Across outcomes, the RAI more consistently differentiated patients at risk for recovery‐ and disposition‐related endpoints, whereas mFI‐5 performance was strongest for select severe complication endpoints. In exploratory analyses of available NSQIP resource‐utilization outcomes, neither frailty index demonstrated significant adjusted associations or clinically meaningful discrimination for 30‐day return to the operating room or readmission.

## 4 Discussion

### 4.1 Summary of Key Findings

In this ACS‐NSQIP cohort of 1871 patients undergoing ESS, frailty was significantly associated with postoperative morbidity and mortality. Increasing RAI categories were independently associated with higher odds of CD II and CD IV complications, eLOS, and NHD, while mFI‐5 primarily identified elevated risk for eLOS, NHD, and CD IV complications. ROC analyses similarly showed that frailty indices provided clinically useful separation of risk; RAI demonstrated stronger or comparable discrimination across all key outcomes evaluated, with the largest separation occurring in resource‐intensive endpoints such as discharge disposition. These findings support frailty as a critical paradigm in ESS outcomes, with the RAI better capturing gradients of vulnerability that drive postoperative recovery.

### 4.2 Findings in Context

These findings reinforce a growing surgical consensus that multidomain frailty instruments outperform comorbidity‐based indices when predicting postoperative recovery rather than discrete complications [30, 31]. Unlike the mFI‐5, which operationalizes frailty largely as accumulated disease burden, the RAI captures physiologic reserve through integration of functional status, physiological limitation, nutritional decline, and systemic vulnerability [22, 26]. Outcomes such as eLOS and NHD are mechanistically linked to these domains, reflecting diminished recovery capacity rather than operative hazard alone [4, 32, 33, 34, 35].

The observed divergence between indices illustrates an important distinction in risk modeling: the magnitude of association does not necessarily translate to discriminatory performance [30, 36]. The mFI‐5 demonstrated meaningful odds ratios for several endpoints; however, its compressed ordinal structure concentrates risk into relatively few strata, permitting substantial probability overlap across patients, a significant statistical limitation for a risk‐stratification index [21, 37]. In contrast, the RAI's broader scale generates more granular vulnerability gradients, enabling clearer separation between patients who are more or less likely to deviate from expected recovery trajectories [22, 29]. This pattern of a strong association but inferior discrimination for comorbidity‐focused indices has been described across higher‐acuity surgical populations, showing that instruments incorporating functional dependence and physiologic decline more reliably predict resource‐intensive outcomes than complication‐centric measures [26, 27, 29, 36]. Collectively, these data support a conceptual shift from comorbidity enumeration toward reserve‐based risk stratification, particularly as postoperative value increasingly hinges on recovery efficiency and maintenance of independence [2, 4].

Within rhinology, where ESS is frequently perceived as lower physiologic stress, these results suggest that frailty operates less as a marker of procedural risk and more as a determinant of postoperative adaptability [16, 25]. The strongest separation occurred in disposition‐related endpoints, representing the clinical expression of depleted reserve, indicating that even moderate frailty carries meaningful implications for perioperative resource utilization [38]. Together, these findings position frailty not merely as a risk modifier but as a framework for anticipating recovery trajectories in an aging surgical population [39, 40].

### 4.3 Practical Implications

From a clinical perspective, these findings support integrating frailty assessment into preoperative ESS workflows to enhance risk stratification, expectation setting and counseling, and perioperative planning [38]. Disposition‐related outcomes are particularly actionable; identifying patients at elevated risk for prolonged hospitalization or NHD enables earlier coordination with case management, anticipatory rehabilitation planning, medication optimization, and caregiver engagement [16, 25, 41].

The RAI may be uniquely well suited for this role given its multidimensionality and weighted metric, which more closely approximates the physiologic reserve required for postoperative recovery after even modest surgical stress [22]. Although the mFI‐5 offers practical advantages in simplicity and feasibility, its narrower focus on comorbidity burden may incompletely capture functional vulnerability, a factor tightly linked to resource utilization and loss of independence. Although the mFI‐5 ostensibly offers convenience through a shortened index, the majority of the RAI's components are routinely documented within electronic medical records [41, 42, 43].

Importantly, frailty screening should not be interpreted as a mechanism to restrict access to ESS when clinically indicated. Rather, it provides a quantitative framework to support shared decision‐making, guide perioperative optimization strategies, and align postoperative resources with anticipated recovery needs [44, 45]. As surgical care increasingly emphasizes value and individualized risk assessment, incorporating multidimensional frailty evaluation may represent a meaningful step toward more patient‐centered perioperative planning [38, 44].

### 4.4 Limitations

This retrospective NSQIP analysis is constrained by registry accuracy and available variables; cognition and granular nutritional metrics are incompletely captured, and the RAI was operationalized administratively rather than at the bedside. NSQIP lacks granular ESS indication data, including disease severity, revision status, tumors, CSF leaks, and epistaxis, as well as imaging and endoscopic findings. Although CPT codes and nonelective status were used, residual confounding by surgical indication remains possible. Follow‐up is limited to 30 days, potentially underestimating longer‐term recovery differences. NSQIP‐defined wound complications, including SSI and dehiscence, were retained as standardized safety measures but should be interpreted cautiously as exploratory endpoints rather than ESS‐specific markers of postoperative sinonasal healing. Finally, low event rates and small counts in the highest frailty strata produced wide confidence intervals, limiting precision for some comparisons.

### 4.5 Future Directions

Future studies should validate these findings in ESS cohorts with richer rhinologic clinical detail, including disease severity, revision status, surgical indication, radiographic burden, and postoperative endoscopic outcomes. Prospective studies should also evaluate bedside frailty screening, such as the RAI‐C, integrated into preoperative rhinology clinic workflows. Moving forward, it will be important to determine whether targeted optimization strategies for frail patients, including prehabilitation, nutritional support, pulmonary optimization, medication review, caregiver engagement, and structured post‐discharge planning, can reduce resource‐intensive outcomes. In addition, comparing RAI and mFI‐5 alongside other commonly used geriatric tools may clarify which approach best balances feasibility with predictive performance in routine rhinology practice. More broadly, standardizing frailty measurement across surgical specialties may reduce fragmentation and improve comparability across studies, health systems, and perioperative care pathways.

### 4.6 Conclusion

Frailty is a clinically meaningful predictor of postoperative outcomes after ESS. Both the RAI and mFI‐5 identified higher‐risk patients; however, the RAI generally demonstrated stronger or comparable discrimination, particularly for recovery‐ and resource‐associated outcomes such as eLOS and NHD, supporting the value of multidomain frailty assessment even in lower‐risk otolaryngologic surgery. Incorporating frailty screening into ESS preoperative evaluation may improve risk counseling, perioperative planning, and resource allocation, enabling more individualized, value‐based surgical care.

## Funding

The authors have nothing to report.

## Conflicts of Interest

The authors declare no conflicts of interest.

## Supporting information




**Supplemental Figure 1**: Flowchart of Patient Selection for Endoscopic Sinus Surgery Cohort.
