# Supplementary material for: A Comparison of Frailty Indices in Predicting Postoperative Outcomes After Endoscopic Sinus Surgery
Source: Int Forum Allergy Rhinol. 2026 Jul 2;16(11):1280–9. doi: 10.1002/alr.70212 (PMC13731157; doi:10.1002/alr.70212)

Supplemental Figure 1. Flowchart of Patient Selection for Endoscopic Sinus Surgery Cohort

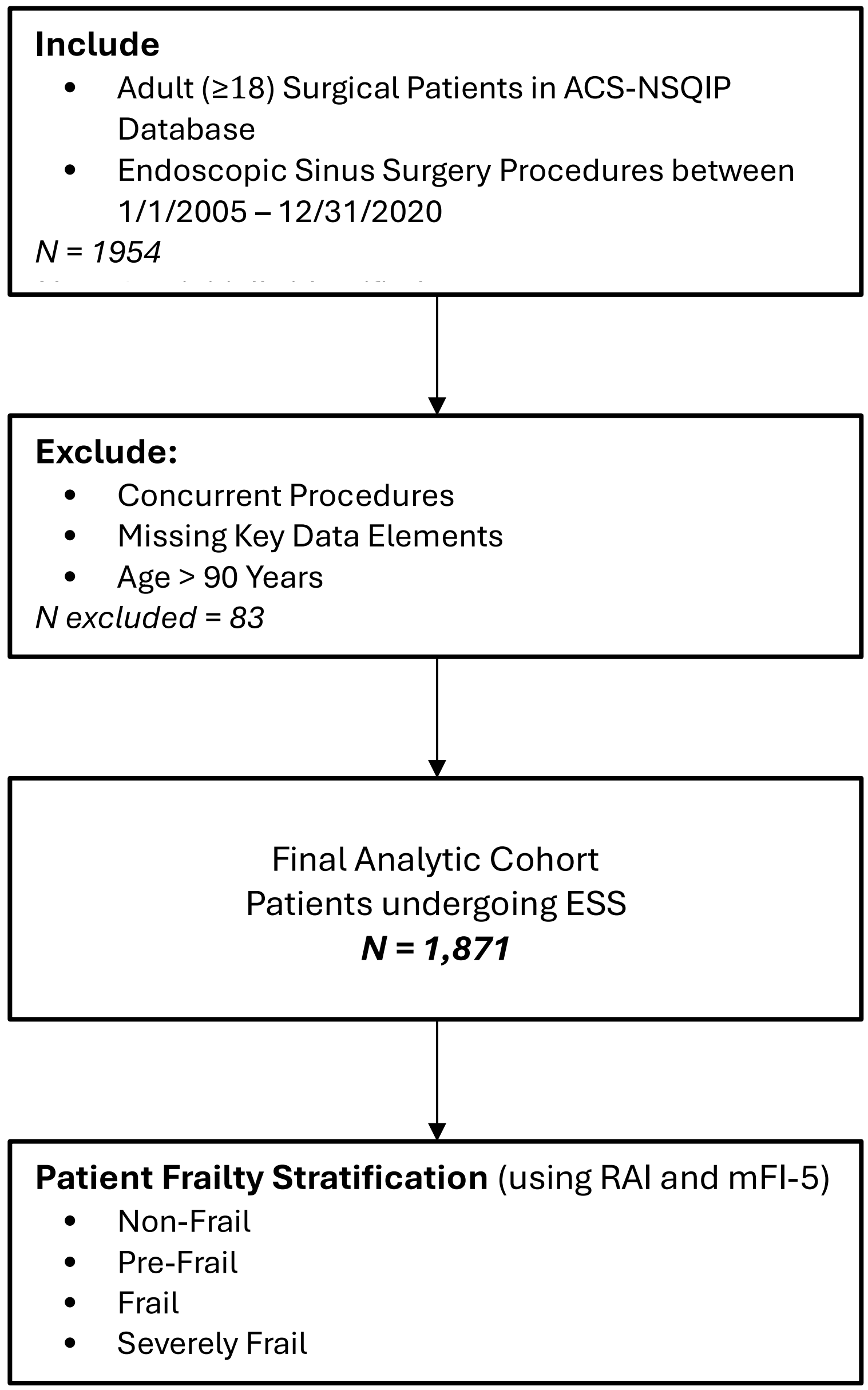

Supplement: Supplementary file 1 [file ALR-16-1280-s001.pdf]
